# Reprogramming of basic metabolic pathways in microbial sepsis: therapeutic targets at last?

**DOI:** 10.15252/emmm.201708712

**Published:** 2018-07-05

**Authors:** Lise Van Wyngene, Jolien Vandewalle, Claude Libert

**Affiliations:** ^1^ Center for Inflammation Research VIB Ghent Belgium; ^2^ Department of Biomedical Molecular Biology Ghent University Ghent Belgium

**Keywords:** hypoxia, inflammation, interventions, metabolic reprogramming, sepsis, Immunology, Metabolism, Microbiology, Virology & Host Pathogen Interaction

## Abstract

Sepsis is a highly lethal and urgent unmet medical need. It is the result of a complex interplay of several pathways, including inflammation, immune activation, hypoxia, and metabolic reprogramming. Specifically, the regulation and the impact of the latter have become better understood in which the highly catabolic status during sepsis and its similarity with starvation responses appear to be essential in the poor prognosis in sepsis. It seems logical that new interventions based on the recognition of new therapeutic targets in the key metabolic pathways should be developed and may have a good chance to penetrate to the bedside. In this review, we concentrate on the pathological changes in metabolism, observed during sepsis, and the presumed underlying mechanisms, with a focus on the level of the organism and the interplay between different organ systems.

**Glossary**
Aerobic glycolysis: A.k.a. Warburg effect: first described by Otto Warburg as a characteristic of cancer cells. Warburg observed that cancer cells generate energy (ATP) through high rates of glycolysis, rather than through oxidative phosphorylation.
Anorexia: Reduced food intake.
Beta‐oxidation: Metabolic pathway in which fatty acids are metabolized to generate energy.
Catabolism: Process that breaks down molecules into smaller units.
Cecal ligation and puncture (CLP) model: One of the most stringent models of sepsis. It involves a combination of three insults: tissue trauma due to laparotomy, necrosis caused by ligation of the cecum, and infection due to the leakage of peritoneal microbial flora into the peritoneum.
Cori cycle: A.k.a. glycose‐lactate cycle: metabolic pathway in which lactate produced by anaerobic glycolysis in the muscles moves to the liver and is converted to glucose, which in turn is metabolized back to lactate in the muscles.
Disease tolerance: Defense strategy limiting the pathologic outcome of infections by preventing tissue damage or ameliorating tissue function without interfering with host's pathogen load.
Electron transport chain: A system of molecules that pass electrons from one to another via redox reactions coupled with the transfer of protons across the inner membrane of mitochondria, creating a proton gradient that drives ATP synthesis.
Epigenome: Heritable chemical changes of the genome, such as DNA methylation or histone modification, that modifies gene expression but does not change the DNA nucleotide sequence itself.
Free fatty acid: A non‐esterified fatty acid, released by the hydrolysis of triglycerides within adipose tissue. Free fatty acids can be used as an immediate source of energy by many organs and can be converted by the liver into ketone bodies.
Glasgow coma scale score: Provides a practical method for assessment of impairment of conscious level based on eye, verbal, and motor criteria.
Gluconeogenesis: Metabolic process in which glucose is formed from smaller precursors, such as amino acids and glycerol.
Glycolysis: Generation of ATP through degradation of glucose, usually associated with anaerobic conditions.
Immunoparalysis: Persistence of a compensatory anti‐inflammatory innate immune response following an insult such as sepsis or trauma.
Ketogenesis: Production of ketone bodies by breaking down fatty acids and ketogenic amino acids. This process supplies the needed energy of certain organs, especially the brain.
Lipolysis: The process of breaking down of lipids into fatty acids and glycerol.
Oxidative phosphorylation: A.k.a. mitochondrial respiration: generation of ATP through the mitochondrial inner membrane via the tricarboxylic acid cycle, whereby oxygen functions as terminal proton acceptor.
Reactive oxygen species (ROS): Derivatives of oxygen generated during mitochondrial oxidative metabolism. A buildup of ROS in cells may cause damage to DNA, RNA and proteins.
Sepsis: A life‐threatening condition that arises when the body's response to an infection leads to tissue and organ damage.
Sequential organ failure assessment (SOFA): Assessment of a patients status, during the stay in an intensive care unit, used to determine the extent of organ dysfunction. The score is based on six different scores (respiratory, cardiovascular, hepatic, coagulation, renal and neurological systems). A higher SOFA score is associated with an increased risk of mortality.
Starvation: Malnutrition following, for example, anorexia, gastrointestinal disease, cancer, and coma. The metabolic response to starvation is mobilization of energy via catabolism of body tissues (muscle, adipose tissue).
Tricyclic acid cycle (TCA): A.k.a. Krebs cycle, is the major energy‐producing pathway and occurs in mitochondria. Short carbon chains derived from sugars, fatty acids, or amino acids are metabolized to yield carbon dioxide, water, and high‐energy phosphate bonds (ATP).

## Introduction: sepsis pathophysiology

Sepsis is a life‐threatening condition resulting from a dysregulated response to infection, with a global burden of at least 19 million cases annually (Singer *et al*, 2016). Classically, the acute phase of sepsis is characterized by an initial strong pro‐inflammatory and innate immune status aimed at eliminating the pathogen. During the later phase of sepsis, the immune system shifts toward an anti‐inflammatory, immune‐suppressive status, resulting in diminished inflammation, and initiation of tissue repair. Based on these insights, many immunomodulatory therapies have been tested in clinical trials in sepsis over the last decades, but no actual novel therapy based on inflammatory or immune pathways has demonstrated survival benefit. The only agent that has gained regulatory approval was activated protein C, but this was later withdrawn from the market due to safety and efficacy concerns (Ranieri *et al*, 2012). Current management of sepsis is supportive rather than curative and is aimed at eradication of the infection, fluid resuscitation to maintain organ perfusion, vasopressor administration to maintain an adequate blood pressure and mechanical support of failing organs. Even in the face of improved technical advances, mortality rates of 20–25% are commonly reported (Marshall, 2014). Increasing rates of antimicrobial resistance and aging of the human population add to the problem and underscore the need for innovative therapeutic strategies.

One of the explanations why so many clinical trials have been disappointing is that sepsis has been considered too much as an inflammatory disease, while recent research suggests an important contribution of coagulation, complement activation, microbiome composition, thermoregulation, circadian rhythm, and metabolism (Cohen *et al*, 2015). Indeed, the pathogenesis of sepsis is clearly influenced by profound changes in metabolic homeostasis. Typical features of sepsis, such as high fever, tachycardia, tachypnea, inflammation, immune activation, phagocytosis, and acute phase reactant production, all require supra‐physiological energy supplies. However, despite their increased nutritional requirements, patients are often unwilling or unable to eat, which creates a problem of energy deficit. Also, one recurrent feature in sepsis is a clear problem of mitochondrial respiration (Protti *et al*, 2007). Biopsies taken from skeletal muscle from deceased sepsis patients found a strong decrease in ATP/ADP ratio (Lee & Hüttemann, 2014).

Similarly, in mice and rats, sepsis led to decreased ATP levels in skeletal muscle and liver (Lee & Hüttemann, 2014). The immediate result is a high catabolic state leading to the breakdown of carbohydrates, lipid, and protein reserves (Englert & Rogers, 2016). Interestingly, a large proteomic and metabolic screen on plasma of sepsis patients identified glucose metabolism and fatty acid beta‐oxidation pathways as being significantly different between sepsis survivors and non‐survivors (Langley *et al*, 2013). In human patients, hyperglycemia is very often observed and occasionally hypoglycemia occurs. A clear increase in L‐lactate is usually seen directly related to poor outcome, besides signs of poor fatty acid oxidation and amino acid catabolism. It is believed that short‐term and mild changes in metabolism can positively modulate immune responses to eliminate pathogens and to protect the host via disease tolerance, that is, a defense strategy that limits the pathologic outcome of infections without interfering directly with the hosts pathogen load (Soares *et al*, 2014). Uncontrolled, severe disturbances of metabolic homeostasis are however detrimental (Balmer & Hess, 2017). Thus, changes in metabolism during sepsis can well be the master regulator pathways that aim to provide energy to overcome the critical situation, but at the same time save energy because of poor nutritional input. How the septic organism copes with this schism, and whether these insights can lead to new therapeutic options, is reviewed in this paper.

## Sepsis and mitochondrial metabolism

A recurrent finding in sepsis is the obvious problem with mitochondrial metabolism (Park & Zmijewski, 2017). Even in septic conditions, glycolysis appears to be perfectly ongoing and pyruvate is abundantly generated in the cytoplasm of cells. Under well‐oxygenated conditions, pyruvate enters the mitochondria via a heterodimeric mitochondrial pyruvate carrier protein (MPC) complex. We are not aware of studies reporting a reduced expression or function of MPC in sepsis. In contrast, the enzyme complex responsible for the next necessary step of pyruvate breakdown is heavily compromised in sepsis: the transformation of pyruvate into acetyl‐CoA and CO_2_. The multiprotein enzyme pyruvate dehydrogenase complex (PDC) is a big, strongly conserved complex that is tightly regulated in activity and is an important sensor of oxygen and a regulator of TCA activity. In sepsis, PDC activity is clearly reduced (Vary, 1996; Nuzzo *et al*, 2015) and several explanations have been provided. PDC needs a co‐factor, thiamin (a.k.a. vitamin B1), and this molecule has been found to be reduced in sepsis samples, but clinical trials using thiamin were rather disappointing (Leite & de Lima, 2016). More likely, PDC loses activity due to phosphorylation by a set of kinases known as pyruvate dehydrogenase kinase, PDKs. There are four such kinases, PDK1 to PDK4. These kinases are regulated by transcription [e.g., by transcription factors such as hypoxia‐inducible factor 1α (HIF‐1α), glucocorticoid receptor (GR), peroxisome proliferator‐activated receptor (PPAR‐α), and others] as well as by other mechanisms (Jeoung, 2015). Researchers have also found other kinases that inhibit PDC activity, notably the mitogen‐activated protein kinase (MAPK) kinases (Park & Jeoung, 2016) and the kinase involved in necroptosis, receptor interacting protein kinase 1 (RIPK3; Yang *et al*, 2018). Obviously, there is a clear point of intersection of different potentially key pathways in sepsis, namely inflammation, hypoxia, and stress. Once inactivated, the PDC complex can return to an active status by means of de‐phosphorylating phosphatases. From an evolutionary point of view, PDC is closely related to α‐ketoglutarate dehydrogenase complex, which is similar in structure, also needs thiamin, and performs de‐carboxylation and addition of CoA to a substrate (forming succinyl CoA from α‐ketoglutarate in the TCA cycle). There are however no reports of changes of this enzyme in sepsis.

Other problems with mitochondria have been reported in relation to sepsis. Several groups have demonstrated that mitochondria display physical damage (Zang *et al*, 2014). Dysfunction of mitochondria in sepsis has been described as being a direct result of small, reactive molecules, produced in the first wave of inflammation, for example NO, CO, and reactive oxygen and nitrogen species, whereby mitochondrial DNA is damaged as well as proteins of the electron transport chain (ETC.; Svistunenko *et al*, 2006). The ETC. multiprotein complexes have also been described as being less abundant in cells from septic patients due to reduced expression (Lee & Hüttemann, 2014). The ETC. serves to transport the electrons generated in the TCA cycle and finally to reduce the terminal electron acceptor O_2_ to water, in coordination with flux of protons from the matrix into the intermembrane space and back to the mitochondrial matrix. In sepsis, complex I and complex II of the ETC. have been found to be less active, but also complex IV has been claimed as a major mitochondrial target (Hüttemann *et al*, 2012b). Phosphorylation of this complex [by inflammatory kinases such as c‐Jun‐N‐terminal kinase (JNK) that enter mitochondria] is thought to lead to physical problems with mitochondria, including leakage of matrix components into the cell, exposing cytochrome C which initiates apoptosis of the cells (Hüttemann *et al*, 2012a). This view is compatible with the observations that in sepsis, mitochondria are less involved in oxidative phosphorylation and more in the induction of apoptosis (Lee & Hüttemann, 2014).

Despite the strong evidence pointing toward mitochondrial dysfunction during sepsis, other studies have shown that mitochondrial function in sepsis is highly variable between organs and over the course of the disease. These statements open the discussion to whether mitochondrial impairment is the major driver of organ failure in septic shock and urges the field to investigate this issue in more detail.

The increased production of lactate in sepsis is thought to be the result of increased glycolysis activity (aerobic glycolysis a.k.a. Warburg effect) and increased reduction of pyruvate, and this is based on several changes, which may be problematic in sepsis. First, there is the reduction in PDC activity, and other issues with mitochondria, as just explained. Second, in sepsis there is cytopathic hypoxia. Several authors have shown that the oxygen levels in tissues during sepsis are rather normal (Fink, 2015) but that there is a reduced capacity of the tissues to utilize O_2_ from the blood (Suetrong & Walley, 2016). Other authors have demonstrated that during a decrease in oxygen delivery to cells (oxygen delivery = cardiac output × O_2_ carrying capacity of blood), normal cells will respond with a production of lactate when oxygen delivery reaches a lower limit of about 400 ml/min. Septic cells will however respond much more sensitive, that is, from a lower limit of about 600 ml/min (Suetrong & Walley, 2016). Third: despite there being no hypoxia *senso stricto*, septic cells will perform a hypoxic response and there is transcriptional increase and activation of HIF1‐α (further described below). This transcription factor will induce several genes, coding for proteins that are involved in glycolysis, namely hexokinase, phosphofructokinase‐1, and most importantly lactate dehydrogenase A (*LDHA*; Marín‐Hernández *et al*, 2009), which converts pyruvate into lactate. Lactate is then pumped into the extracellular space by increased expression of MCT4 (coded by the *SLC16A3* gene). Fourth, lactate is transported in the blood and, as a reduced metabolite, can be used as an energy source by different tissues, such as cardiac muscle. Already four decades ago, it was shown that in sepsis, the oxidation of lactate to pyruvate is defective, most likely because due to aberrations of two mitochondrial shuttle systems which transport the protons (derived from the oxidation of lactate) into the mitochondria (Jones, 1974). Fifth, under physiological conditions and during physical activities, lactate released into the blood will be taken up by the liver and used as a substrate to form glucose by a process called gluconeogenesis. This cycle of glucose to lactate catabolism followed by new glucose production from lactate is called the Cori cycle, after Gerty and Carl Cori, rewarded by the Noble Award in 1947. As shown by Clemens *et al*, in a rat sepsis model (the cecal ligation and puncture (CLP) model; Dejager *et al*, 2011), this lactate‐based gluconeogenesis in liver is severely reduced (Clemens *et al*, 1983). The mechanism behind this defect in gluconeogenesis is unknown, but could in part be explained by the fact that, the first step that oxidizes lactate to pyruvate, again requires one of these defective mitochondrial shuttles. Moreover, even if pyruvate is formed, it will have to be transformed to phosphoenolpyruvate (PEP) via a step performed in mitochondria and again involving an oxaloacetate‐malate‐oxaloacetate shuttle, leading to oxaloacetate in the cytoplasm and its de‐carboxylation to PEP via the critical enzyme PEPCK (coded by *PCK1*). Several studies have shown that PCK1 expression is significantly decreased in the liver in sepsis (Deutschman *et al*, 1993).

In conclusion, based on these mechanisms, in sepsis, there is a clear increase in production of lactate, and the major pathways to remove lactate are blocked. Lactate is a typical biomarker of bad prognosis in sepsis, since lactate levels strongly correlate with disease severity, morbidity, and mortality in sepsis (Nichol *et al*, 2010; Rishu *et al*, 2013). The degree of lactate clearance in a septic patient, during the first 6 h of sepsis treatment, is highly associated with survival in sepsis (Nguyen *et al*, 2004). Lactate may lead to lactic acidosis (serum pH < 7.35), which results in reduced glucose uptake by the brain, leading to coma, and induction of heart arrhythmias and heart failure (Suetrong & Walley, 2016). A low Glasgow Coma Scale score and cardiovascular failure are both factors influencing the Sequential [Sepsis‐related] Organ Failure Assessment (SOFA) score, which is a score reflecting how severely compromised different organ systems are in sepsis (Singer *et al*, 2016). Severe lactic acidosis (pH < 7.2) is associated with a mortality rate of about 50%, and even no surviving patients has been reported with severe lactic acidosis with a pH under 7.0 (Kimmoun *et al*, 2015). Lactate has been reported not only as an important intermediate in metabolic processes and as a cause of pH decline, but also as a molecule actively influencing inflammation. For example, lactate boosts Toll‐like receptor‐4 (TLR4), that is, the major receptor of LPS, signaling, and NF‐κB‐mediated gene transcription (Samuvel *et al*, 2009) and promotes HMGB1 release (Yang *et al*, 2014) in macrophages, suggesting that lactate promotes inflammation.

The problems with mitochondrial function also impact fatty acid and protein catabolism, as described further.

## Sepsis and carbohydrate metabolism

### Hyperglycemia

**Figure 1:**
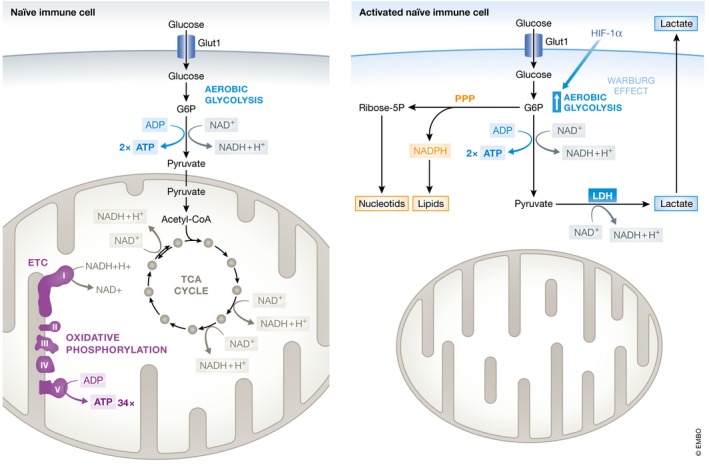
Impact of sepsis on carbohydrate metabolism Naïve immune cells rely on glycolysis and oxidative phosphorylation as the main metabolic pathways to generate ATP. Glycolysis is the catabolic process in which glucose is converted into pyruvate. Extracellular glucose is imported into cells by GLUT1 (*SLC2A1* gene). Subsequent intracellular processing of glucose yields two pyruvate molecules and two ATP molecules by a series of enzymatic reactions. At sufficient O_2_ tension, pyruvate is imported in mitochondria and converted into acetyl‐coA and enters the tricarboxylic acid cycle (TCA cycle, a.k.a. Kreb's cycle), after which each pyruvate yields 17 ATP molecules by the electron transport chain (ETC) and oxidative phosphorylation. Upon activation of immune cells, the glycolysis pathway is upregulated under the control of HIF‐1α. The metabolic pathway shifts from oxidative phosphorylation to aerobic glycolysis, also referred to as the Warburg effect, to meet the increased energy demand in activated immune cells. Despite the presence of abundant O_2_ in the environment, glucose is then directly metabolized into lactate. Although energetically less favorable (glycolysis generates 2 molecules of ATP out of 1 molecule glucose, whereas oxidative phosphorylation provides 36 ATP molecules), aerobic glycolysis allows higher velocity of glycolysis and thus faster ATP production, as well as provides important precursors for the synthesis of lipids, amino acids, and nucleotides required for proliferation. Interfering with aerobic glycolysis in immune cells has been shown to undermine their anti‐infectious activities, highlighting the importance of this altered metabolism in activated immune cells.

**Figure 2:**
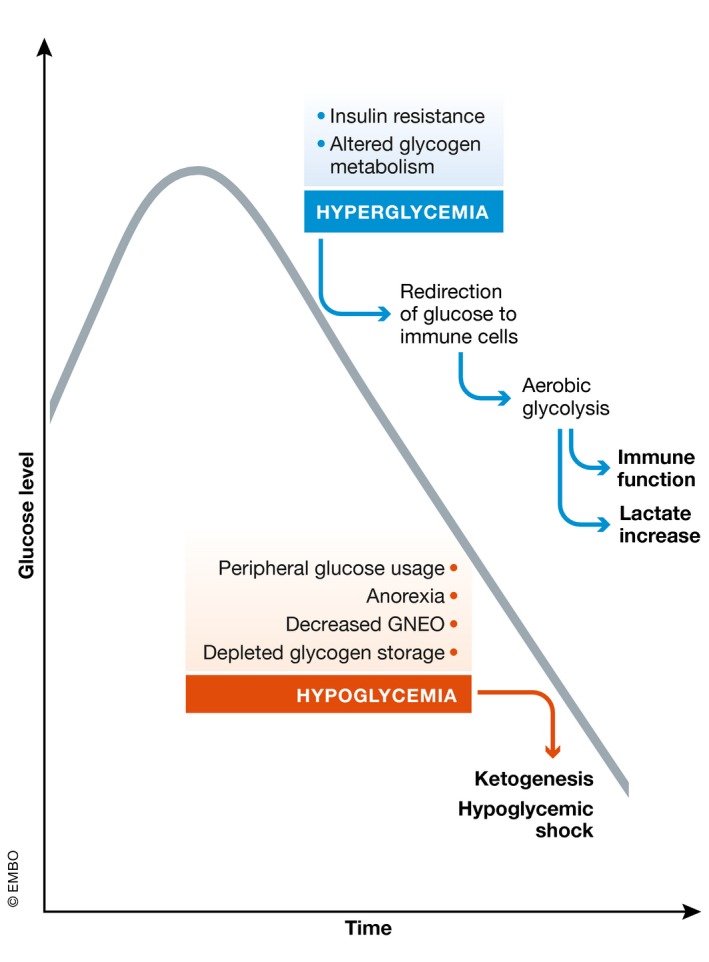
The pathogenesis of sepsis is associated with a deregulation in glucose metabolism Both high and low glucose levels correlate with sepsis severity. Initially, a hyperglycemic response is observed in both animal models and in human patients, presumably resulting from insulin resistance and altered glycogen metabolism. This allows redirection of glucose to immune cells supporting aerobic glycolysis and thus immune function, and also leads to lactate production. In later stages, hypoglycemia could be observed as a consequence of several factors. In contrast to animal models, hypoglycemia is less frequently observed in human patients.

The pathogenesis of sepsis is associated with a deregulation in glucose metabolism resulting in a metabolic and energetic failure (Fig 1). Hyperglycemia is one of the most prevalent metabolic derangements in sepsis patients, presumably resulting from altered glycogen metabolism and profound insulin resistance (Englert & Rogers, 2016). From an evolutionary perspective, hyperglycemia is thought to be desirable as it provides glucose to cells that do not rely on insulin for glucose uptake, such as neurons and leukocytes (Marik & Bellomo, 2013). Redirection of glucose to immune cells allows meeting their rapid division as well as their bio‐energetic demands during inflammation. Indeed, naïve cells rely mainly on oxidative phosphorylation and beta‐oxidation of free fatty acids as energy sources, whereas activated immune cells rely mainly on aerobic glycolysis, also referred to as the Warburg effect (Cheng *et al*, 2016; Fig 2). Although energetically less favorable in terms of ATP production, aerobic glycolysis is adopted by activated immune cells because it provides biosynthetic intermediates that are essential for the synthesis of nucleotides, lipids, and amino acids. These are important to support proliferation and the synthesis of inflammatory molecules. For instance, glucose 6‐phosphate (G6P), generated by the first step in glycolysis, can feed into the pentose phosphate pathway to support nucleotide synthesis. This pathway also generates NADPH which is essential for lipid synthesis. Furthermore, an adequate immune response requires rapid energy production and aerobic glycolysis provides ATP swiftly. Oxidative phosphorylation requires mitochondrial biogenesis, which is a complex and slow process, whereas glycolysis could be induced rapidly by inducing glycolytic enzymes (Loftus & Finlay, 2016; O'Neill *et al*, 2016). Conversion of glucose into lactate allows high rates of glycolysis providing rapid ATP production and lactate can also be used as a highly reduced (H‐rich) tri‐carbon fuel. Finally, because of suboptimal ETC. function, mitochondrial TCA‐mediated respiration in sepsis leads to the production of dangerous reactive oxygen species (ROS; Arulkumaran *et al*, 2016), a process which can also be prevented by switching to glycolysis, which generates ATP without ROS production and this might be a form of damage control.

Glycolysis has been shown to be crucial for a number of immune cell functions in several activated immune cells in order to meet their increased energy needs. For example, glycolysis is specifically required for phagocytosis and cytokine production in pro‐inflammatory (M1) macrophages (Yang *et al*, 2014; Jha *et al*, 2015), for cytokine production in T cells (Chang *et al*, 2013), for antibody production in B cells (Caro‐Maldonado *et al*, 2014) and for antigen presentation in dendritic cells (Everts *et al*, 2012, 2014). The increased serum lactate levels during sepsis hence could also originate from activated immune cells undergoing aerobic glycolysis (Cheng *et al*, 2014b).

From the above‐mentioned studies, it is clear that there is a close link between metabolism and inflammatory responses. If it is true that high lactate levels would be derived from white blood cells, therapeutic modulation of leukocyte metabolism could potentially represent a novel therapeutic approach in sepsis. Appropriate inflammatory responses are essential for pathogen elimination, but excessive inflammation may result in overstimulation, organ damage, and even death.

One key mediator of aerobic glycolysis is the transcription factor HIF‐1α (Corcoran & O'Neill, 2016). HIF‐1α regulates the expression of aerobic glycolysis‐related genes (such as glucose transporter 1 (GLUT1, encoded by *SLC2A1*), *LDHA,* and *PDK4,* as well as inflammatory genes, notably interleukin‐1β (*IL1B* gene; Corcoran & O'Neill, 2016) and inducible NO synthase (*NOS2* gene). Under hypoxic conditions, but also by stimulation by bacterial products [e.g., lipopolysaccharide, LPS, of Gram‐negative bacteria (Nishi *et al*, 2008)], and pro‐inflammatory cytokines [e.g., TNF (Regueira *et al*, 2009)] under normoxic conditions, HIF‐1α protein is stabilized and accumulates in cells. Conditional knock‐out of HIF‐1α in myeloid cells was found to decrease inflammatory cytokine production and to protect animals against endotoxemia, an experimental model of sepsis (Peyssonnaux *et al*, 2007). Functional work with HIF‐1α KO mice in real bacterial sepsis models has not been performed so far.

Another important mediator in aerobic glycolysis is pyruvate kinase isoenzyme M2 (PKM2), which acts as a co‐activator for HIF‐1α and is also involved in a classical metabolic step, namely the last step of glycolysis, that is, the de‐phosphorylation of phosphoenolpyruvate to pyruvate. It is activated in hypoxia but also induced after LPS stimulation. Knockdown of PKM2 decreases lactate production and cytokine release both *in vitro* and *in vivo* leading to protection of mice from lethal endotoxemia and sepsis (Yang *et al*, 2014). Impairing the transcriptional activity of the PKM2/HIF‐1α complex by use of small molecules polarizes M1 macrophages into an M2 phenotype, leading to reduced IL‐1β and induced IL‐10 production. However, counteracting the inflammatory response with these small molecules in an *Salmonella typhimurium* model interferes with the bacterial killing capacity leading to increased bacterial dissemination (Palsson‐Mcdermott *et al*, 2015).

Also mammalian target of rapamycin (mTOR) has been described as an important regulator of HIF‐1α activity. mTOR activity is induced by hypoxia, growth factors, and a number of ligand‐receptor systems, and leads to increased translation of a number of key mRNAs, including HIF‐1α mRNA (Cheng *et al*, 2014a). mTOR is negatively involved in the function of regulatory T cells (Treg), because increased glycolysis in peripheral Treg cells may impair their lineage and survival integrity (Wei *et al*, 2016). Inhibition of the mTOR pathway by metformin decreases cytokine production in a *Candida albicans* infection model, leading to increased fungal outgrowth and decreased survival of mice in this infection model (Cheng *et al*, 2016).

We thus see here a number of examples that show that inhibition of cytokine release, via manipulation of metabolic pathways, undermines the activities of essential immune cells, which blunts the proper clearage of the infection. So, it is important to limit exaggerated immune and inflammatory responses during sepsis, while ensuring a minimal amount of immunity for the clearing of the ongoing infection.

Besides targeting HIF‐1α, PKM2, mTOR, or other similar central coordinating factors, another strategy to target glycolysis is with 2‐deoxy‐D‐glucose (2‐DG). Hexokinase (HK) is the initial and rate‐limiting enzyme in glycolysis). HK converts 2‐DG to phosphorylated 2‐DG (2‐DG‐6p), which inhibits HK activity thereby inhibiting glycolysis. *In vitro*, 2‐DG suppresses HIF‐1α and its targets IL‐1β and HMGB1 in activated macrophages (Tannahill *et al*, 2013; Yang *et al*, 2014). In T cells, blocking glycolysis with 2‐DG leads to a differentiation of Th17 cells into Treg cells (Shi *et al*, 2011). *In vivo*, 2‐DG significantly improves survival in both the LPS (Wang *et al*, 2016) and CLP (Zheng *et al*, 2017) model of sepsis by downregulating lactate and inflammatory cytokine production. In contrast, 2‐DG conferred no protection in a poly(I:C)—induced systemic inflammation model (Wang *et al*, 2016).

Patients that survive the acute hyperinflammatory phase of sepsis remain at an increased risk for secondary infections, and consequently late stage mortality (Wang *et al*, 2014). Understanding the mechanisms that underlie immunoparalysis in sepsis is also crucial for the identification of novel therapeutic approaches in sepsis. Immunotolerant leukocytes show broad metabolic defects at the level of both glycolysis and oxidative metabolism. One group has demonstrated that restoring the ability of immunotolerant leukocytes to mount a glycolytic response by treatment with IFN‐γ leads to an increased cytokine production and might represent a promising novel therapeutic approach to revert the immunotolerant state of sepsis (Cheng *et al*, 2016). A phase III clinical trial evaluating the effect of IFN‐γ on sepsis‐induced immunoparalysis (NCT01649921) has been conducted, but results have not yet been reported. The same group showed that aerobic glycolysis is also important for trained immunity, that is, the memory characteristics of the innate immune system. Pretreatment of mice with the β‐glucan component of *C. albicans* induces a non‐specific protection against infections, such as with *Staphylococcus aureus*. However, mice with a myeloid‐cell‐specific defect in HIF‐1α are unable to mount a trained immunity against *S. aureus* and succumb to the infection (Cheng *et al*, 2014a).

### Hypoglycemia

In later stages, sepsis can also be characterized by hypoglycemia. In preclinical animal models, for example, mouse endotoxemia or mouse CLP, hypoglycemia is inevitable and linked with severity (Drechsler *et al*, 2015). Also in human patients, spontaneous hypoglycemia can be observed (Miller *et al*, 1980; Romijn *et al*, 1990; Rattarasarn, 1997; Singanayagam *et al*, 2009) but occurs less frequently. The fact that hypoglycemia occurs in mouse sepsis models and is less observed in human sepsis, may be based on (i) the relatively big surface to volume ratio in mice compared to humans, by which mice therefore lose heat much faster than men, and have to compensate this by a higher heart rate and metabolic turnover, and (ii) because humans are fed with glucose and other nutrients in the ICU. The mechanisms leading to hypoglycemia are not well understood. Depleted glycogen storages, increased peripheral usage of glucose, anorexia (Wang *et al*, 2016; Weis *et al*, 2017), and/or decreased gluconeogenesis (Dendoncker & Libert, 2017; Weis *et al*, 2017) may all be contributing factors.

Acute infections are associated with the development of anorexia, that is, an aspect of sickness behavior characterized by reduced food intake and thereby reduced blood glucose levels. It has recently been shown that this fasting metabolic state is crucial for survival of bacterial sepsis, whereas it may be detrimental for viral inflammation (Wang *et al*, 2016). Prolonged fasting results in hypoglycemia accompanied by lipolysis and ketogenesis, that is, the generation of ketone bodies. Ketone bodies [there are three common such ketone bodies, namely acetone, acetoacetate, and β‐hydroxybutyrate (BHB)] have been shown to confer resistance to reactive oxygen species (ROS)‐mediated damage. By supplementing glucose during bacterial sepsis, ketogenesis is suppressed, leading to inhibition of these ketone body‐mediated ROS adaptation pathways. This mechanism seems to be required for preventing ROS‐induced neuronal damage in bacterial inflammation, whereas it is dispensable in case of viral inflammation (Wang *et al*, 2016). Next to ketone bodies, alternative pathways that control ROS production, the release of ROS into the cytoplasm, the interaction of ROS with specific substrates such as kinases, and the detoxification of ROS can play important roles during sepsis pathophysiology, as was recently reviewed comprehensively by Kozlov *et al* (2017). In contrast, the study of Weis *et al* has shown that liver glucose production in response to bacterial infection is essential to establish disease tolerance. The authors suggest that liver gluconeogenesis is required to prevent lethal hypoglycemia in response to acute infection. According to this study, gluconeogenesis in the liver is hampered during sepsis due to iron‐driven oxidative inhibition of glucose‐6‐phosphatase (Weis *et al*, 2017). Presumably, anorexia is important to establish disease tolerance to systemic bacterial infections, and this could explain why this behavior is conserved from humans to insects (Wang *et al*, 2016). However, the blood glucose levels need to be maintained within a physiological range compatible with host survival and might thus not fall below a certain threshold (Chervonsky, 2017).

### Control of glucose levels in sepsis: controversy

Both high (Gunst & Van den Berghe, 2016) and low glucose (Singanayagam *et al*, 2009; Krinsley *et al*, 2011) levels correlate with poor outcome in sepsis patients. Severe hyperglycemia has been shown to induce, that is, mitochondrial damage, endothelial dysfunction, acute kidney injury, liver dysfunction, muscle weakness, and long‐term neurocognitive impairment (Gunst & Van den Berghe, 2016). Severe hypoglycemia, in contrast, has been demonstrated to induce, for example, sudden cardiac arrest by inducing ischemic or depolarization/repolarization changes, neurocognitive impairment (acute coma), endothelial dysfunction, blood coagulation abnormalities, and increased inflammation (Kalra *et al*, 2013).

Three pioneer randomized controlled trials on tight glucose control found that control of glucose levels with insulin improved the outcome in sepsis patients as compared to control groups (Van den Berghe *et al*, 2001, 2006; Vlasselaers *et al*, 2009). These studies have led to a change in standard of care and have implemented control of glycemic levels to prevent excessive hyperglycemia (Gunst & Van den Berghe, 2016). However, there have been some concerns about the risk of developing severe hypoglycemia, the difficulty of achieving normoglycemia in ICU patients, and the contradicting results of some follow‐up studies (Marik, 2016). Therefore, a new clinical trial to test the value of tight glycemic control was conducted, namely the Normoglycemia in Intensive Care Evaluation–Survival Using Glucose Algorithm Regulation (NICE‐SUGAR) trial (Riske *et al*, 2009). The NICE‐SUGAR trial found that intensive glucose control in fact increased mortality, suggesting that slightly elevated glucose levels are rather beneficial and that glycemic control in ICU patients should be abandoned. It should be noted that in the NICE‐SUGAR study, the control group was also treated with insulin to keep glycemic levels between 140 and 180 mg/dl, instead of the uncontrolled hyperglycemia (up to 215 mg/dl) in the first studies (Gunst & Van den Berghe, 2016). It thus remains unclear how to manage glucose levels in ICU patients, but it seems that safe, effective glucose control may be advantageous over tight glycemic control.

## Sepsis and fatty acid metabolism

**Figure 3:**
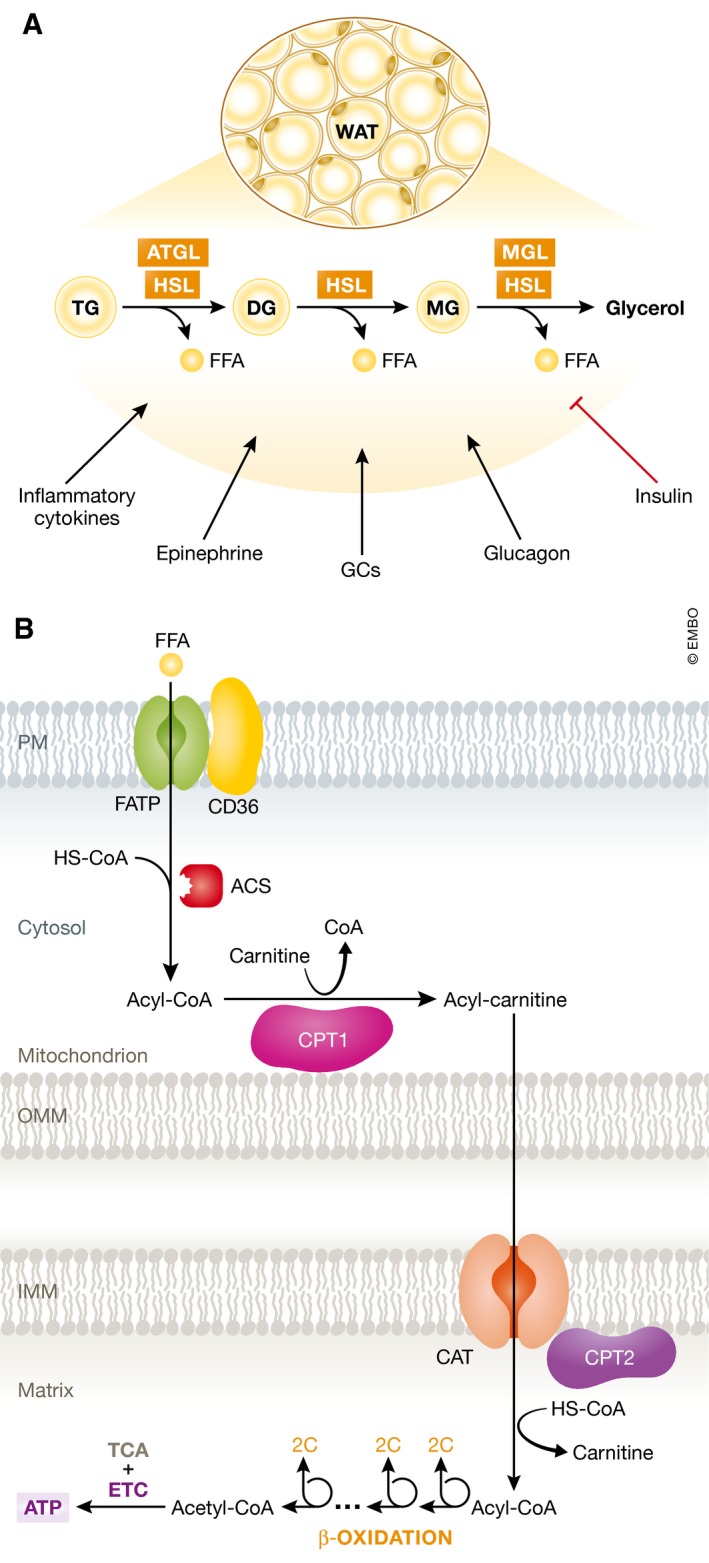
An overview of lipolysis and lipid oxidation in healthy conditions (A) Lipolysis is the hydrolytic conversion of triglycerides (TG) into glycerol and free fatty acids (FFAs) and is most abundant in white and brown adipose tissue. This process is tightly regulated by glucagon, (nor)epinephrine, and other hormones, but also by pro‐inflammatory cytokines. Hydrolysis of the ester bonds between long‐chain fatty acids and the glycerol backbone is executed by lipases. Up to now, three enzymes have been implicated in performing the complete hydrolysis of TG into FFAs and glycerol: adipose triglyceride lipase (ATGL), hormone sensitive lipase (HSL), and monoglyceride lipase (MGL). The FFAs are released into the blood stream and can be taken up by peripheral organs to produce energy via mitochondrial β‐oxidation. (B) Fatty acid β‐oxidation is a multistep process breaking down fatty acids in the mitochondria of the cell to produce acetyl‐CoA, which can be used by the tricarboxylic acid (TCA) cycle to produce ATP. In brief, FFAs are transported across the cell membrane by members of the FATP transporter family. Once inside the cytosol, the FFA is coupled to coenzyme A (CoA) by acyl‐CoA synthetase (ACS) and shuttled across the inner mitochondrial membrane by carnitine palmitoyltransferase II (CPT2) and the carnitine acyltransferase (CAT) after being coupled to carnitine by carnitine palmitoyltransferase I (CPT1). In the mitochondrial matrix, β‐oxidation is conducted by cleaving two carbon molecules in every oxidation cycle to form acetyl‐CoA. The cycle is repeated until the complete fatty acid has been reduced to acetyl‐CoA, which is subsequently enters the TCA cycle.

Substantial activation of the immune system during sepsis, combined with the inability of most patients to keep up adequate nutrition, induces a starvation response in which next to glycolysis, energy needs are also supplied by lipid mobilization and oxidation (Jorgen *et al*, 1982; Wolowczuk *et al*, 2008). The storage of lipids as triglycerides (TGs) in adipose tissue comprises the bodies’ largest endogenous energy supply, allowing the release of fatty acids to become crucial during states of acutely increased energy needs (Cahill, 1970; Rittig *et al*, 2016). When energy demands are elevated, the body responds by upregulating lipolysis in adipose tissue, converting TGs into glycerol and free fatty acids (FFAs), which are subsequently released into the bloodstream (Cahill, 1970). FFAs can be taken up by peripheral organs and converted into energy by means of the β‐oxidation pathway followed by the TCA cycle (Fig 3). The average fat storage in a young human adult is 10–20 kg, the variation depending on gender, age, and fitness. The human glycogen reserve, in contrast, consists only of approximately 400 g, of which 300 g is largely unavailable and sealed within the skeletal muscles, reinforcing the importance of fat as potential energy storage (Sloan *et al*, 1973).

### Lipolysis

Next to a deregulated glucose metabolism, the pathogenesis of sepsis is also known to influence fatty acid metabolism. Decades ago, it has already been described that lipolysis is upregulated in white adipose tissue (WAT) during the early phase of endotoxemia and sepsis (Forse *et al*, 1987), a finding that was more recently confirmed by several other studies (Wellhoener *et al*, 2011; Ilias *et al*, 2014; Rittig *et al*, 2016). Consequently, plasma TGs and FFA levels have been found to be increased up to 4‐fold in septic patients compared to healthy individuals (Scholl *et al*, 1984; Lanza‐Jacoby & Tabares, 1990; Nogueira *et al*, 2008). The molecular mechanisms behind this upregulation of lipolysis have hardly been addressed. One study showed that LPS infusion in human subjects led to an increased phosphorylation of hormone sensitive lipase (HSL) at Ser^650^, a modification known to activate the enzyme (Rittig *et al*, 2016). HSL is one of the best described lipases (encoded by the *LIPE* gene) and could, together with the other important enzyme adipose triglyceride lipase (ATGL, *PNPLA2* gene), be involved in the (patho)physiological lipolysis in white adipose tissue (WAT). Also, cAMP‐dependent protein kinase A (PKA)‐dependent phosphorylation of perilipin 1 (PLIN1) is a decisive step in the activation of lipolysis (Choi *et al*, 2010) and was found to be elevated after LPS infusion (Rittig *et al*, 2016). This suggests that increased PKA activity might be one of the driving forces behind the increased lipolysis. Sepsis and endotoxemia are known to cause an acute increase in stress hormones such as (nor)epinephrine, growth hormone, glucagon, and cortisol, all of them being typical pro‐lipolytic hormones, which indeed stimulate lipolysis by direct stimulation of lipolysis in fat cells (Fong *et al*, 1990; Bloesch *et al*, 1993). Moreover, insulin resistance is a well‐established phenomenon in septic patients and insulin is a potent inhibitor of lipolysis (Choi *et al*, 2010). Eventually, all these mechanisms are likely to converge into an increase in the amounts of FFAs released by lipolysis into the bloodstream in sepsis.

### β‐oxidation

Oxidation of FFAs provides a crucial source of energy during conditions of energy needs, starvation, or acute inflammation, including sepsis (Askanazi *et al*, 1980; Jorgen *et al*, 1982). However, the pro‐inflammatory status of this pathology is known to cause a dysregulation in the breakdown of FFAs in a wide variety of organs. A whole blood genome‐wide expression profiling in patients with septic shock showed that the expression of genes belonging to the PPAR‐α signaling pathway was significantly downregulated in children with a more severe disease status (Wong *et al*, 2009). PPAR‐α is a member of the nuclear receptor family and the most important transcription factor involved in the transcriptional regulation of genes coding for proteins involved in the β‐oxidation pathway (Hiukka *et al*, 2010).

**Figure 4:**
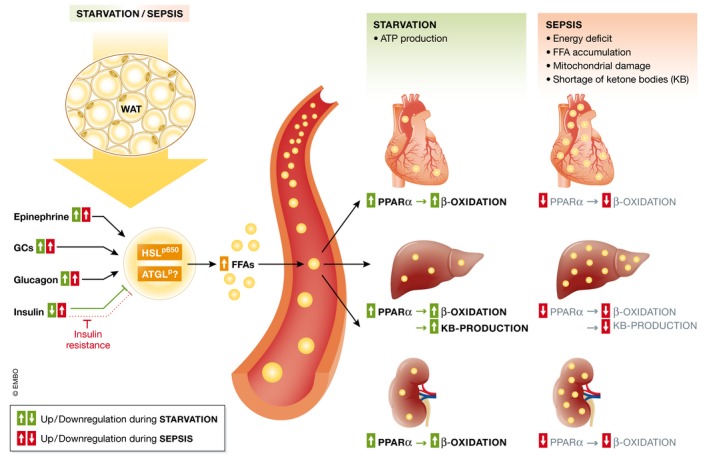
Lipolysis, fatty acid oxidation, and ketogenesis in sepsis Sepsis is associated with the development of an anorectic response since patients are often unwilling or unable to eat. During a normal starvation response and during sepsis, lipolysis in white and brown adipose tissue is being upregulated by several pro‐lipolytic signals. The inhibitory effect on lipolysis of insulin, which is upregulated in sepsis due to high glucose levels, is however absent due to insulin resistance. Free fatty acids (FFAs) in the blood are upregulated in both conditions and can be taken up by peripheral organs to produce energy. The increased FFA levels activate and upregulate the expression of PPAR‐α, the main transcription factor responsible for the induction of genes involved in the β‐oxidation of fatty acids and the production of ketone bodies (KBs). During sepsis, PPAR‐α levels are downregulated and the breakdown of fatty acids through β‐oxidation is compromised, causing FFAs to accumulate in organs such as the liver, heart, and kidney, but also in the blood. Overall, the deficits in FFA breakdown during sepsis cause a shortage of energy and lipotoxicity and mitochondrial damage due to FFA accumulation. Green represents the normal starvation response, and red represents the response during sepsis.

Standage *et al* (2012) demonstrated more recently that the downregulated expression of PPAR‐α dependent genes was due to a reduced PPAR‐α expression in whole blood samples, a decrease which was associated with decreased survival and increased tissue bacterial load in an experimental polymicrobial septic mouse model. Furthermore, using bone marrow transplantation experiments, it was proven that disease severity is primarily determined by organ tissue PPAR‐α expression and not by hematopoietic immune cell PPAR‐α (Standage *et al*, 2012). Downregulation of PPAR‐α expression levels during infection has also been demonstrated in the liver, heart, and kidney (Beigneux *et al*, 2000; Feingold *et al*, 2008; Drosatos *et al*, 2011). In the heart, it was shown that LPS injection caused a decrease in PPAR‐α as well as in PPAR‐y co‐activator (PGC)‐1α. PGC‐1α is a major transcriptional co‐factor of PPAR‐α, and its downregulation thus leads to decreased expression of several PPAR‐α target genes and a reduction in the rate of FFA acid β‐oxidation in cardiomyocytes. As a consequence of the reduced oxidative breakdown of FFAs, severe organ damage may occur due to (i) energy deprivation, (ii) FFA accumulation and lipotoxicity, and (iii) mitochondrial dysfunction and endoplasmic reticulum (ER) stress (Fig 4).

#### Energy deprivation

Each of the previously specified organs is highly metabolically active in order to provide adequate energy to perform vital homeostatic functions within the body. When increased metabolic needs during acute inflammation are not met, in part due to mitochondrial dysfunction, life‐threatening organ dysfunction can develop, a recurrent feature of septic shock (Carré & Singer, 2008). It has been hypothesized that restoration of the FFA oxidation in specific organs could protect against organ dysfunction and potentially lead to increased survival in sepsis. In a zymosan‐induced (sterile) peritonitis model, it was shown that the upregulation of β‐oxidation of FFAs was associated with the resolution of inflammation (Fujieda *et al*, 2013). Furthermore, in macrophages and adipocytes, enhanced FFA oxidation led to reduced ER stress and ROS damage. It was also demonstrated that increased β‐oxidation improved insulin resistance by reducing the triglyceride accumulation and the expression of inflammatory cytokines, two factors that have been shown to reduce the sensitivity to insulin (Malandrino *et al*, 2015). Another study demonstrated that intervention in the LPS‐induced JNK signaling pathway restored PPAR‐α expression in the heart and prevented LPS‐induced heart dysfunction (Drosatos *et al*, 2011).

#### Lipotoxicity

The combination of increased lipolysis and deficits in the oxidation of FFAs can lead to the accumulation of these FFAs and subsequently to lipid‐induced toxicity. A proteomic and metabolic screen on plasma of septic patients identified several FFAs to be significantly upregulated in the blood of non‐survivors compared to survivors (Langley *et al*, 2013). Lipotoxicity is defined as a metabolic syndrome resulting from the accumulation of lipid intermediates in non‐adipose tissue and can lead to cellular dysfunction and cell death (Engin, 2017). Under normal conditions, lipids are stored primarily in adipocytes, with only a minimal presence in other tissues (Tontonoz & Spiegelman, 2008). When an overload of FFAs is present due to increased lipolysis and/or decreases in β‐oxidation, lipid deposits can be formed in peripheral organs. Presumably, this accumulation of fatty acids is not toxic at first; in fact, it can be seen as some sort of defense mechanism against the excess of circulating FFAs (Brindley *et al*, 2010). By taking up a part of the fatty acid overload, metabolically active organs such as the liver may protect more sensitive organs including the lungs from the toxic side effects of high FFA levels. Clinically, this phenomenon is addressed as *steatosis* and has been identified in liver, kidney, and heart after the onset of endotoxemia and sepsis (Zager *et al*, 2005; Rossi *et al*, 2007; Koskinas *et al*, 2008). When lipids continue to accumulate, certain lipid metabolites such as diacylglycerol (DAG), ceramide, and saturated fatty acids reach a critical level that could potentially be harmful to the tissue cells. It has been accepted that excess of lipids are ultimately steered toward non‐oxidative pathways which results in the formation of toxic lipid species that cause mitochondrial dysfunction (Bugger & Abel, 2008), modify cellular signaling(Yang & Barouch, 2007) and increase apoptosis (i.e., “lipoapoptosis”; Unger & Orci, 2002). More recently, toxic lipid species have been associated with the induction of ferroptosis, an alternative type of programmed cell death dependent on iron. The role of ferroptosis as a cell death mechanism contributing to organ damage in sepsis has been hardly explored. Nevertheless, ferroptosis has been implicated in acute kidney failure and could be an important alternative cell death pathway during sepsis (Linkermann *et al*, 2014; Müller *et al*, 2017; Wenzel *et al*, 2017). The precise contribution and impact of each of above‐mentioned altered cellular processes have not been properly delineated and seem to depend on lipid composition and cell type (Ghosh & Rodrigues, 2006). For a more detailed discussion on lipotoxicity, we refer to a number of excellent recent reviews (Wende & Abel, 2010; Ertunc & Hotamisligil, 2016; Engin, 2017).

#### Mitochondrial dysfunction

Mitochondrial dysfunction due to the fatty acid overload can be caused by the increased production of ROS (Schönfeld & Wojtczak, 2008), the uncoupling of the oxidative phosphorylation (Rial *et al*, 2010) and the permeabilization of the outer mitochondrial membrane (Listenberger & Schaffer, 2002). As stated before, it has been described that systemic inflammation can affect mitochondria by several mechanisms such as the generation of excess amounts of nitric oxide, carbon monoxide and ROS which directly inhibit mitochondrial function and damage the lipid membrane (Bauer & Pannen, 2009; Larsen *et al*, 2012). It seems plausible that the excess amount of fatty acids might contribute to the development of mitochondrial dysfunction during sepsis. Lipotoxicity has also been linked to ER stress and excessive activation of the unfolded protein response (UPR; Cunha *et al*, 2008; Baldwin *et al*, 2012). Many ER stress responses converge with inflammatory pathways via the activation of inflammatory kinases such as c‐Jun‐N‐terminal kinase (JNK) and the I‐kappa‐B‐kinase complex (IKK) and the activation of the inflammasome (Hu *et al*, 2006; Nakamura *et al*, 2010). Saturated fatty acids, including palmitate, can bind to TRL4 and induce a MyD88‐dependent signaling to eventually activate the inflammasome and NF‐κB signaling pathways (Lee *et al*, 2001). Moreover, ceramide, which is produced from long‐chain saturated fatty acids, has been shown to be directly associated with the induction of insulin resistance and lipoapoptosis (Kanety *et al*, 1996; Paz *et al*, 1997; Brookheart *et al*, 2009).

### Therapeutic intervention

It is highly possible that the overload of FFAs, caused by aberrations in the fatty acid metabolism, contributes to the pathogenesis of sepsis and that interventions to prevent this accumulation could potentially ameliorate outcomes in sepsis. To avoid the accumulation and toxic effects of FFAs, one could target several pathways of the fatty acid metabolism: lipolysis, β‐oxidation, or lipogenesis.

Pharmacological inhibition of one of the major lipases, adipose triglyceride lipase (ATGL), was successful in correcting high‐fat diet‐induced insulin resistance and hepatosteatosis in mice (Schweiger *et al*, 2017). Several potent inhibitors that are directed toward the other major lipase, HSL, have recently been produced and might provide an attractive alternative to treat sepsis (Ogiyama *et al*, 2017).

Pharmacological prevention of PPAR‐α downregulation shows potential since inhibition of JNK restored PPAR‐α protein levels and was enough to prevent myocardial dysfunction after infection(Drosatos *et al*, 2011; Drosatos & Schulze, 2013). Also, interventions that target co‐factors or downstream targets of PPAR‐α could possibly contribute to improved oxidation of FFAs and enhanced energy production. However, extensive research into the mechanisms by which PPAR‐α is downregulated in different organs and other changes within this metabolic pathway are still needed to provide a complete view on possible druggable targets.

Another option could be to interfere before PPAR‐α is downregulated by actively stimulating PPAR‐α by the means of agonists. Indeed, mice on a fenofibrate‐loaded diet were found to show improved survival and repression of the inflammatory response in infection models (Tancevski *et al*, 2014). Such a therapy, of course, is purely preventive, and indeed rarely relevant in a clinical setting. One could argue that increasing the capacity of WAT to store lipids through adipogenesis by PPAR‐γ stimulation might decrease ectopic lipid overload (Gema *et al*, 2007). The PPAR‐γ agonist thiazolidinedione (TZD) was shown to improve peripheral insulin sensitivity and clearance of TGs in type 2 diabetes (Mayerson *et al*, 2002), while ameliorating murine polymicrobial sepsis by reducing the pro‐inflammatory signal transducer and activator of transcription‐1 (STAT‐1) signaling pathway (Ferreira *et al*, 2014). Moreover, several studies reported a role for PPAR‐γ in inducing mitochondrial biogenesis (Bolten *et al*, 2007; Pardo *et al*, 2011), a process that has been associated with survival during early sepsis (Carré *et al*, 2010). Combinatorial PPAR‐α and PPAR‐γ stimulation, while actively repressing lipolysis during the early stages of sepsis could represent an interesting alternative approach. Prospective clinical studies toward the use of fibrates and TZDs in infectious conditions have been conducted, but are however still insufficient and should be revisited (Fujita *et al*, 2006; Boggild *et al*, 2009).

In conclusion, an activation of lipolysis, associated with a break on FFA β‐oxidation, due to reduced activity of PPAR‐α and PGC‐1α and poor function of mitochondria, appears very critical in sepsis and is open to several key therapeutic interventions. The precise genes that suffer from reduced transcriptional activity of PPAR‐α and the impact on poor FFA oxidation have hardly been investigated, but the ones that coordinate cellular (*CD36*) and mitochondrial FFA import (*SLC25A20*,* CPT1A*,* CPT1B*), as well as those involved in the real oxidation pathway, appear logical priorities to study.

## Sepsis and ketogenesis

As discussed above, sepsis leads to a fasting response accompanied by a pro‐lipolytic status with increased lipolysis and ketogenesis (Wang *et al*, 2016). Synthesis of the three known ketone bodies is tightly controlled by PPAR‐α and is mainly conducted by mitochondria of liver cells. Next to conferring resistance against ROS‐mediated damage, KBs are also an important source of energy for brain and skeletal muscle in times of high metabolic activity and limited glucose availability such as fasting and exercise (Randle *et al*, 1964). The production of KBs thus ensures that not all energy which is contained in FFAs is entirely degraded to acetyl‐CoA and further to CO_2_ and H_2_O in the liver TCA cycle, but that other organs, with much less FFA oxidation capacity can profit from the FFAs released by lipolysis. Aberrations in ketone metabolism during sepsis have been described a long time ago by several research groups (Felig *et al*, 1971; Flatt & Blackburn, 1974; Wannemacher *et al*, 1979). This suggests that the reduced production of KBs could lead to the increased breakdown of muscle protein, a universally accepted consequence of sepsis. Moreover, a recent study has shown that in PPAR‐α‐deficient mice LPS‐induced endotoxemia was lethal due to the absence of ketogenesis (Wang *et al*, 2016). Thus ketogenesis appears essential in the defense to sepsis but may be inadequately active in sepsis. Also, BHB was shown to block NLRP3‐inflammasome‐mediated inflammation not through the classical starvation‐regulated mechanisms such as AMP‐activated protein kinase (AMPK) or ROS, but by mechanically preventing the K^+^ efflux and reducing oligomerization of inflammasome components (Youm *et al*, 2015). Furthermore, BHB administration to mice exposed to high doses of fish oil was protective against acute liver failure and the lipotoxic effect of peroxidized fatty acids (Pawlak *et al*, 2015). In 1994, a study by Beylot *et al* demonstrated the inhibitory effect of BHB on lipolysis in septic patients, unfortunately, mortality rates were not considered during this trial (Beylot *et al*, 1994). Since NLRP3 and high levels of FFAs have been implicated in the pathogenesis of sepsis (Jin *et al*, 2017), administration of ketone bodies might thus be considered as a valuable novel therapeutic direction.

## Sepsis and amino acid metabolism

In sepsis, a general catabolic status is observed. As we described above, a starvation‐like status leads to breakdown of carbohydrate and fat reserves, but also protein is degraded in several organs. Proteolysis, the trimming of proteins into smaller polypeptides and amino acids (AAs) has been best documented in skeletal muscle. The signals that lead to extensive muscle wasting or the proteases involved are poorly described in this condition. Similar to glycogen breakdown in liver and lipolysis in white adipose tissue, proteolysis is most likely the result of hormonal regulation, with glucagon and glucocorticoids being potential candidate regulators, as well as proteasomal proteases and inflammatory stimuli (Biolo *et al*, 1997). The precise reason for the increased proteolysis in sepsis fits into a general reshuffling of energy‐rich molecules, as well as an increased need of AAs in the liver to sustain the acute phase response (Hasselgren *et al*, 1988). Several amino acids are also known to play an important role in inflammatory cells. For example, glutamine is an important precursor for peptide and protein synthesis supporting cytokine production. Glutamine is also required for purine and pyrimidine and thus nucleic acid and nucleotide synthesis allowing proliferation of immune cells. Arginine can be converted into nitric oxide by nitric oxide synthase, which is then released by M1 macrophages.

All AAs are found to be increased in muscle cells, by proteolysis, and new amino acids seem to be formed due to catabolic chemical reactions (Su *et al*, 2015). Also in plasma, most amino acids are highly altered. Certain amino acids, especially taurine, are found to play an important role in predicting the severity and outcome in sepsis (Su *et al*, 2015). Supplementation of amino acids, such as glutamine, arginine, and taurine, during sepsis showed positive outcomes such as reduced length of stay and reduced number of secondary infections (Arts *et al*, 2016).

**Figure 5:**
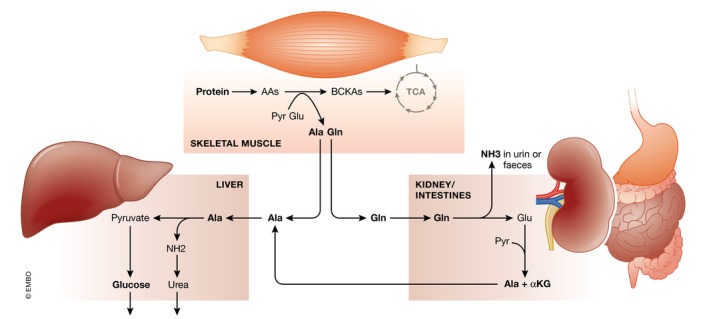
Overview of protein catabolism in sepsis In sepsis conditions, catabolism of proteins in skeletal muscle is a recurrent feature, but the main regulators are still not identified. Branched‐chain amino acids (AAs) are oxidized to branched‐chain keto acids (BCKAs), which can be used in the TCA cycle. Glutamine (Gln) and alanine (Ala) find their way to kidney and intestine and liver. In the former two, Gln is de‐aminated to glutamate (Glu) and ammonia (NH_3_), which is removed. Glu and pyruvate can yield Ala and α‐ketoglutaric acid (αKG), which can enter the TCA cycle. Ala is mainly used as a gluconeogenic substrate and is transformed to pyruvate, whereby the NH_2_ group is removed via the urea cycle. During liver failure, ammonia may leak into the blood, leading to brain damage and coma.

Specifically the branched‐chain amino acids (BCAAs), such as alanine, leucine, and isoleucine, are of interest. These AAs can function as acceptors of keto‐groups from pyruvate and glutamate, leading to glutamine and alanine. These AAs can enter the blood and can be used as energy‐rich AAs by other organs. Organs that are classically considered as targets are the kidney, intestine, and liver (Fig 5). In kidney and intestine, glutamine can be de‐aminated to glutamate, which can enter the TCA cycle via α‐ketoglutarate, potentially leading to the formation of alanine. Under normal conditions, the NH_3_ that is released during glutamine conversion is removed via urine or the intestinal lumen. Glutamine metabolism in the liver is low, especially because the liver has no big capacity to deal with NH_3_. Alanine, produced in the muscle, kidney, and intestine is transported to the liver, where it is oxidized and de‐aminated into pyruvate, which can subsequently enter the TCA cycle. Depending on the degree of hypoxia, pyruvate can be reduced to lactate or lead to glucose via gluconeogenesis. In septic patients, an increase in gluconeogenesis due to elevated alanine uptake in liver would be preferential, but, as stated above, gluconeogenesis is compromised in sepsis, which might therefore increase alanine levels in the blood of septic patients as observed by Langley *et al* (2013). Increases in ammonia in sepsis blood are observed in case of severe hepatic failure (Nesseler *et al*, 2012), a condition that is estimated to occur in about 20% of septic shock patients. Under such conditions, liver failure leads to a multitude of problems, such as bad hepatic capture and transport of bilirubin and bile salts, but also increased ammonia, resulting from a poor urea cycle. This latter, in essence a direct result of proteolysis, leads to hepatic encephalopathy, that is, mental disorientation of patients, a common finding, and coma (Felipo & Butterworth, 2002).

## Epigenetic‐metabolic link in sepsis

Epigenetics is defined as a “stably heritable phenotype that does not involve alterations in the DNA sequence, but chemical changes within the chromatin” (Berger *et al*, 2009). These changes include posttranslational modification of histone proteins (such as acetylation, methylation, phosphorylation, and ubiquitination), DNA methylation, and more recently also transient chromatin modifications and miRNAs (Natoli, 2010). Histone modifications are completed by specific sets of enzymes adding or removing different categories of posttranslational modifications, thereby directly modulating the chromatic structure and gene expression (Bannister & Kouzarides, 2011; Verdin & Ott, 2015). Interestingly, the activity of these different enzymes is depending on the presence of specific metabolites, causing chromatin modifications to be directly coupled to the metabolic state of the cell. It is now generally accepted that cells evolutionary acquired sensing mechanisms to detect changes in nutrients and accommodate the transcriptional program via epigenetic changes (Etchegaray & Mostoslavsky, 2016). On the other hand, key metabolic‐regulating genes are under the control of epigenetic machineries, creating a feedback loop that could potentially result into a vicious circle during conditions where metabolic pathways are severely altered.

### Epigenetic changes during sepsis

Bomsztyk *et al* (2015) were the first to investigate changes in histone modifications on a whole‐organ level in sepsis‐related multiple organs dysfunction syndrome (MODS). They showed that 6 h after the onset of infection, major epigenetic changes were occurring in several organs. Moreover, the decrease in gene expression of key genes involved in endothelial function was associated with a decrease in permissive histone modifications (acetylation), while repressive marks (methylation) were unchanged, suggesting that intervention early into the disease to preserve these permissive marks could help to keep endothelial integrity intact. A major limitation of this study is the fact that within every organ, many different cell types are found, each with their own epigenome. More importantly, during sepsis, there is an extensive infiltration of immune cells, making a cell‐specific approach crucial.

Acetylation of lysine residues of histones and other proteins is one of the best known posttranslational modifications and is performed by KATs (lysine acetyl transferases). The activity of these enzymes relies on the availability of acetyl‐CoA, an intermediary metabolite produced mainly from β‐oxidation of fatty acids and the oxidative de‐carboxylation of pyruvate by PDC (Lee & Workman, 2007; Etchegaray & Mostoslavsky, 2016). In nutrient‐favorable conditions, high acetyl‐CoA levels trigger the regulation of specific genes involved in growth and proliferation through histone acetylation, resulting in a more permissive chromatin configuration (Wellen *et al*, 2009; Cai *et al*, 2011). As described above, both acetyl‐CoA‐producing pathways are reduced in sepsis with the oxidation of fatty acids being downregulated and pyruvate being shuttled toward aerobic glycolysis for the production of lactate. As a consequence, it is possible that less acetyl‐CoA is available for acetylation of proteins which could explain the reduction in permissive histone modifications observed by Bomsztyk *et al* (2015). Interestingly, a clear link between the reduction in FFA oxidation, consequent low acetyl‐CoA levels, and low expression of neovascular genes by suboptimal histone acetylation has been shown in mouse models in the recent past (Schoors *et al*, 2015).

### Histone deacetylases in sepsis

Another group of enzymes, involved in the removal of acetylation groups, histone deacetylases (HDACs), might be of interest during the pathology of sepsis. Several histone deacetylase inhibitors (HDACi) have been tested as negative regulators of the expression of important immune receptors and antimicrobial pathways in immune cells. It was shown in multiple studies that HDACi can improve the outcome after septic shock by reducing the cytokine storm, confirming the hypothesis that these inhibitors have potential as sepsis therapeutics (Li *et al*, 2009; Zhang *et al*, 2010; Roger *et al*, 2011). However, broad HDACi have also been associated with impaired bacterial clearance and a more recent study suggested that specific HDAC6 inhibition might improve responses during sepsis, while preserving important microbicidal actions (Zhao *et al*, 2014). The catalytic activity of SIRT6, a member of the class III HDACs, was shown to be increased by certain FFAs up to 35‐fold *in vitro* (Feldman *et al*, 2013). It has been hypothesized that in nutrient conditions where FFAs are increased, SIRT6 activity could repress the transcription of HIF‐1α driven glycolytic genes by removing permissive acetylated histone marks (Zhong *et al*, 2010). During serious infections where the increase in lipolysis causes FFAs levels to rise, this mechanism could contribute to the reduction in histone acetylation and alter the glucose metabolism. On the other hand, SIRT6 acts as a tumor suppressor in cancer cells by inhibition of aerobic glycolysis (Zhong *et al*, 2010; Sebastián *et al*, 2012). This raises the question whether SIRT6 activation could also have potential as a druggable target during severe acute inflammatory conditions.

Another SIRT enzyme that provides a link between metabolism and inflammation is SIRT1. This deacetylase is activated by AMPK, a crucial metabolic sensor that is activated under conditions of elevated energy demand characterized by an increase in the AMP/ATP ratio (Lan *et al*, 2008). SIRT1 has been shown to directly interact with and deacetylate PGC‐1α, as discussed above, a potent co‐factor of many transcription factors involved in energy expenditure such as GR, PPAR‐α, and PPAR‐γ, thereby enhancing their transcriptional activity (Vega *et al*, 2000; Rodgers *et al*, 2005). Pharmacological activation of SIRT1 shows potential for the treatment of sepsis since PGC‐1α expression was found to be severely downregulated in muscle of septic mice, while SIRT1 activation proved to increase survival in the CLP sepsis model (Rocheteau *et al*, 2015; Opal *et al*, 2016). Whether this reduction in mortality is caused in part by improved PGC‐1α transcriptional activity is still unexplored.

It is clear that the impaired energy metabolism in sepsis can drive many epigenetic changes that could exacerbate the dysfunction of inflammatory and metabolic pathways. With more extensive research into the variable epigenetics during sepsis and the possible consequences, we could be paving the way to a new kind of drugs for care of septic patients.

## Conclusion

In a healthy organism, energy expenditure and energy income are in balance. During extensive exercise, the consumption of O_2_ by the TCA cycle exceeds the available amounts, and the resulting hypoxia leads to a mainly HIF‐1α coordinated closure of the mitochondrial import of pyruvate, and an increased production of lactate, which can be consumed elsewhere in the body. The toxic lactate causes muscle cramps, enforcing the end of the exercise. During starvation, that is, when no or limited amounts of food enters the system, a prolonged imbalance of the energy homeostasis is created and in essence two systems are initiated to cause rearrangements of the metabolism. First, hormones (glucagon, epinephrine, norepinephrine, glucocorticoids), sensing low metabolic concentrations, coordinate a starvation response, leading to the release of energy‐rich molecules from the resources, for example, glucose from glycogen, AAs from protein and FFAs and glycerol from TGs. The AAs will lead to glucose via gluconeogenesis in the liver, a process strongly coordinated by glucocorticoids and the GR, while several of the FFAs act as ligands for another nuclear receptor PPAR‐α. These receptors are transcription factors and induce genes that are essential in the gluconeogenesis and β‐oxidation processes, respectively. This is a system of high efficiency and strongly stimulated by the transcriptional co‐factor PGC1‐α. Second, the amounts of mitochondria, well‐known as the organelles where TCA cycle and FFA β‐oxidation occur, are increased by a process of mitochondrial biogenesis, again by the stimulating action of PGC1‐α.

**Figure 6:**
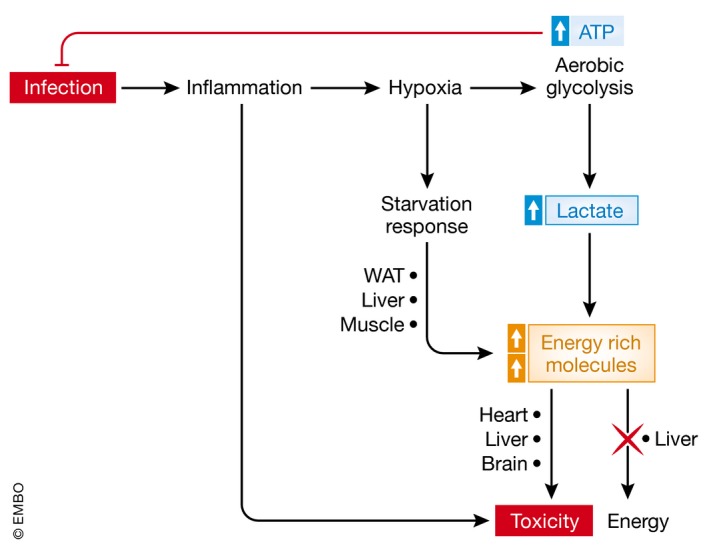
The toxic consequences of metabolic reprogramming in sepsis Infection is the start of sepsis. It leads to direct tissue damage and to inflammation, which in turn leads to hypoxia, which is essential to allow white blood cells (WBCs) to produce fast ATP from glucose and act fast on the infectious agents. The hypoxic response also leads to mobilization of energy‐rich molecules such as lactate and fatty acids, which however can also lead to toxicity, when over abundant.

In sepsis, the energy balance is clearly disturbed. There is energy needed, but patients are unwilling or incapable to eat and a starvation response develops. Due to the infection, an inflammation and immune response develop, and as a consequence a HIF‐1α signature is seen (Fig 6). This leads to a limited mitochondrial function, which may serve several goals. First, inflammation leads to mitochondrial damage, so limiting the importance of these organelles in ATP production seems logical. Second, the metabolism of glucose shifts to aerobic glycolysis, which may be of interest for a more efficient function of white blood cells. Third, some authors suggest that the reduction in mitochondrial respiration under such conditions is a conserved pathway of limiting energy expenditure, leading to a metabolic reprogramming, as is observed during hibernation in several mammalian taxa. Of course, neither humans nor mice are hibernating species, so the importance of these pathways may be questioned. Nevertheless, the danger of a reduced mitochondrial activity is obvious: Most of the energy‐rich molecules produced from the energy stores need active mitochondria to be properly consumed by cells. It is likely that the systemic aspect of sepsis forms the major hurdle of this strategy, because mitochondrial function in sepsis seems to be failing in all tissues, and as a consequence, lactate, FFAs, and other catabolic products accumulate and cause tissue damage and death.

Although it is clear that there is very significant metabolic reprogramming in sepsis, besides the activation of other complex systems (inflammation, coagulation, complement activation, hypoxia response), and given the fact that these pathways all influence one another, it is hard to conclude how, where, and when the metabolic pathways that are calling for therapeutic modulation have to be addressed in a safe and effective way. Based on this overview of the literature, it is our opinion that three early pathways deserve special attention, namely (i) the generation of lactate by the increased HIF‐stimulated glycolysis, (ii) the accumulation of free fatty acids in the blood, by the decreased ability of tissues to oxidize them via beta‐oxidation, and (iii) the decreased generation of ketone bodies by the liver. Since the liver also appears to be undergoing these metabolic rearrangements, and based on the availability of liver‐targeting approaches in today's pharmacology, this organ could be the best option to study in preclinical models of sepsis.

## Conflict of interest

The authors declare that they have no conflict of interest.

## For more information

(i) https://clinicaltrials.gov/ct2/show/NCT01649921?term=NCT01649921&rank=1

**Pending issues**

(i) Failure of all clinical sepsis trials is likely caused due to patient heterogeneity: stratification is the key.
(ii) Studies directed toward the ideal sepsis animal model.
(iii) Studies elucidating sepsis as an inflammatory versus metabolic disorder and identification of key target organs.
